# Supplementary material for: Substrate transport and anion permeation proceed through distinct pathways in glutamate transporters
Source: eLife. 2017 Jun 1;6:e25850. doi: 10.7554/eLife.25850 (PMC5472439; doi:10.7554/eLife.25850)
Supplement: Supplementary file 1. — DOI: http://dx.doi.org/10.7554/eLife.25850.017 [file elife-25850-supp1.docx]

Supplementary file 1

**Substrate transport and anion permeation proceed through distinct pathways in glutamate transporters**

Mary Hongying Cheng *et al*

**Table 1. Summary of MD simulations**

|  | *Run #* | Run Identifier | Duration (ns) | Initial conformation | Voltage  kcal/(mol.Å.e) |
| --- | --- | --- | --- | --- | --- |
| Glt_Ph_ | *1* | cMD_iOFS | 100 | 3V8G | 0.0 |
|  | *2* | tMD1_*i*OFS→IFS | 10 | 6 ns of *run* 1 | 0.1 |
|  | *3* | tMD2_*i*OFS→IFS | 10 | 6 ns of *run 1* | -0.1 |
|  | *4* | tMD3_*i*OFS→IFS | 10 | 6 ns of *run* 1 | 0.3 |
|  | *5* | tMD4_*i*OFS→IFS | 10 | 6 ns of *run* 1 | -0.3 |
|  | *6* | tMD5_*i*OFS→IFS | 10 | 6 ns of *run* 1 | 0.0 |
|  | *7* | tMD6_iOFS→IFS | 10 | 6 ns of *run* 1 | 0.0 |
|  | *8* | cMD1_*iCh*S | 100 | 7.5 ns of *run 6* | 0.0 |
|  | *9* | cMD2_*iCh*S | 100 | 7.5 ns of *run 6* | 0.0 |
|  | *10* | cMD3_*iCh*S | 100 | 6 ns of *run* 8 | -0.1 |
|  | *11* | cMD4_*iCh*S | 100 | 6 ns of *run* 8 | -0.3 |
|  | *12* | cMD5_*iCh*S | 100 | 6 ns of *run* 8 | 0.1 |
|  | *13* | cMD6_*iCh*S | 100 | 6 ns of *run* 8 | 0.3 |
|  | *14* | Meta_*iCh*S | 20 | 6 ns of *run* 8 | 0.0 |
|  | *15* | Meta_S65 | 20 | 100 ns of *run*1 | 0.0 |
|  | *16* | ABF_*iCh*S | 100 | 100 ns of *run*10 | 0.0 |
| EAAT1 | *17* | cMD1_EAAT1_*i*OFS | 100 | EAAT1_*i*OFS | -0.1 |
|  | *18* | cMD2_EAAT1_*i*OFS | 100 |  | 0.1 |
|  | *19* | cMD1_EAAT1_*iCh*S | 100 | EAAT1_*iCh*S | -0.1 |
|  | *20* | cMD2_EAAT1_*iCh*S | 100 |  | 0.1 |
| R477C | *21* | cMD1_R477C_*iCh*S | 50 | R477C of EAAT1_*iChS* | -0.1 |
|  | *22* | cMD2_R477C_*iCh*S | 50 |  | 0.1 |

cMD: conventional MD simulation tMD: targeted MD simulations

Meta: Metadynamics simulations ABF: adaptive biasing force calculations

*i*OFS: intermediate outward-facing state *iCh*S: intermediate channeling state
